# The effect of circular soil biosolarization treatment on the physiology, metabolomics, and microbiome of tomato plants under certain abiotic stresses

**DOI:** 10.3389/fpls.2022.1009956

**Published:** 2022-11-08

**Authors:** Zechariah Haber, María del Mar Rubio Wilhelmi, Jesus D. Fernández-Bayo, Duff R. Harrold, James J. Stapleton, David Toubiana, Jean S. VanderGheynst, Eduardo Blumwald, Christopher W. Simmons, Nir Sade, Yigal Achmon

**Affiliations:** ^1^ School of Plant Sciences and Food Security, Tel Aviv University, Tel Aviv, Israel; ^2^ Department of Plant Sciences, University of California, Davis, CA, United States; ^3^ Department of Food Science and Technology, University of California, Davis, CA, United States; ^4^ Statewide Integrated Pest Management Program, University of California Kearney Agricultural Research and Extension Center, Parlier, CA, United States; ^5^ College of Engineering, University of Massachusetts Dartmouth, Dartmouth, MA, United States; ^6^ Biotechnology and Food Engineering, Guangdong Technion - Israel Institute of Technology, Shantou, Guangdong, China; ^7^ Faculty of Biotechnology and Food Engineering, Technion - Israel Institute of Technology, Haifa, Israel; ^8^ Guangdong Provincial Key Laboratory of Materials and Technologies for Energy Conversion, Guangdong Technion - Israel Institute of Technology, Shantou, Guangdong, China

**Keywords:** rhizosphere microbiome, plant metabolome, abiotic stress, sustainable agriculture, organic waste management, soil solarization, anaerobic soil disinfestation, circular economy

## Abstract

Soil biosolarization (SBS) is an alternative technique for soil pest control to standard techniques such as soil fumigation and soil solarization (SS). By using both solar heating and fermentation of organic amendments, faster and more effective control of soilborne pathogens can be achieved. A circular economy may be created by using the residues of a given crop as organic amendments to biosolarize fields that produce that crop, which is termed circular soil biosolarization (CSBS). In this study, CSBS was employed by biosolarizing soil with amended tomato pomace (TP) residues and examining its impact on tomato cropping under conditions of abiotic stresses, specifically high salinity and nitrogen deficiency. The results showed that in the absence of abiotic stress, CSBS can benefit plant physiological performance, growth and yield relative to SS. Moreover, CSBS significantly mitigated the impacts of abiotic stress conditions. The results also showed that CSBS impacted the soil microbiome and plant metabolome. *Mycoplana* and *Kaistobacter* genera were found to be positively correlated with benefits to tomato plants health under abiotic stress conditions. Conversely, the relative abundance of the orders RB41, MND1, and the family Ellin6075 and were negatively correlated with tomato plants health. Moreover, several metabolites were significantly affected in plants grown in SS- and CSBS-treated soils under abiotic stress conditions. The metabolite xylonic acid isomer was found to be significantly negatively correlated with tomato plants health performance across all treatments. These findings improve understanding of the interactions between CSBS, soil ecology, and crop physiology under abiotic stress conditions.

## Introduction

As the anthropogenic burden on agricultural systems keeps increasing, the need for new sustainable agricultural techniques increases as well. The massive use of modern disruptive techniques in agriculture such as chemical fertilization, fertigation, excessive irrigation, use of chemical pesticides and others is causing both biotic stresses (invasive species, resistant pathogens) and abiotic stresses (soil salinity, heavy metal pollution, nitrogen deficiency, soil erosion and more (Sade et al., 2018; Goswami and Suresh, 2020)) in the soil and on crops. Soils salinity is a rapidly growing global problem (Sade et al., 2020) that necessitates sustainable solutions to increase crop growth in harsh conditions for the maintaining of global food security. Looking for alternative nitrogen sources is also an important mission as chemical fertilizers are a major environmental problem (Savci, 2012). One of the techniques that is being used to mitigate these problems is soil solarization (SS). SS was established as an alternative to chemical soil fumigation to treat a broad spectrum of soil borne pathogens (DeVay and Katan, 1991). SS is done by placing a clear plastic cover over the crop rows ahead of crop planting. The idea behind the technique is to use the resulting heat caused by the greenhouse effect to elevate the soil temperature to a level that can kill a large share of the soil borne pathogens and weeds (Stapleton, 2000). Although SS is a sustainable and environmentally friendly technique that is currently being used around the world (mainly for growing strawberries and other high value crops (Chamorro et al., 2015; Katan, 2015; Oldfield et al., 2017)) it has several pitfalls that prevent it from becoming more popular among farmers. The pitfalls are coming from its passive nature, as it is completely dependent on the available solar radiation, which demands a long duration of treatment and has a limited ability to control soil-borne pathogens beneath the soil surface (Achmon et al., 2017). To overcome these problems a modification of SS is being studied and implemented in the form of soil biosolarization (SBS). In SBS, besides the covering with transparent plastic sheets, additional organic matter (OM) is added to the soil to accelerate the pathogens’ inactivation process by adding biological and chemical pressures on them (Achmon et al., 2017; Hestmark et al., 2019; Achmon et al., 2020). Recent studies done on circular SBS (CSBS, a variety of the SBS technique in which the amendments to the soil are the output residues of the same crop that is established after the SBS treatment thereby using the “closing the loop” concept) together with industrial tomato pomace (TP) residues (e.g. the waste residues of the tomato processing industry) showed promising results in terms of inactivation of weeds and pathogens (Achmon et al., 2016; Achmon et al., 2017) and also in terms of tomato crop growth and health (Achmon et al., 2018). Additionally, more emphasis was given in recent studies to the impact that SBS has on the soil microbial population (e.g. soil microbiome) (Fernández-Bayo et al., 2019; Achmon et al., 2020; Shea et al., 2022). In these studies it was observed that SBS can alter the soil microbiome, mainly due to the combination of elevated quantity of organic matter, higher temperature and anaerobic conditions that serve as natural selective forces for an adaptive microbial community (Achmon et al., 2020). Yet, the impact that these changes in the microbiome have on the consecutive crop is still unknown. At the same time it is observed that the microbiome under SBS treatment also have impacts on the soil chemical composition by addition of products from the microbial metabolism such as volatile fatty acids (VFAs) and other organic compounds (Hestmark et al., 2019; Fernandez-Bayo et al., 2020; Liang et al., 2022). These compounds in turn serve both as selective forces and pathogens’ suppressors, but on the other hand can also have some phytotoxic effect on the consecutive crop growth (Achmon et al., 2016; Achmon et al., 2018). SBS and CSBS have also the advantage over standard SS in that they can be used as an additional venue to valorize organic wastes such as food waste (Spang et al., 2019; Liang et al., 2022) and agricultural waste (Fernández-Bayo et al., 2017; Hestmark et al., 2019; Fernandez-Bayo et al., 2020). While SBS looks as a promising sustainable agricultural technique that can have multiple advantages, the impact on consecutive crop plants and its ability to mitigate the adverse effect of abiotic stress are still unknown. In this study the impact of CSBS with TP on consecutive tomato crop under salinity and nitrogen deficiency abiotic stresses was investigated. The study was aimed to elucidate the complex interaction between the soil microbiome of the CSBS treatment and the tomato plants metabolome (e.g. the collection of primary metabolites of the plant) under abiotic stressors compared to the SS traditional treatment.

## Materials and methods

### CSBS field experiment

Mesocosms (3.8 L plastic soil growth bags) were prepared using soil collected from the field site (UC Davis Plant Pathology Research Farm (Davis, CA, USA; 38.521028, –121.760755; elevation 18.5 m above sea level)). The field soil was sandy clay loam (47%, 27% and 26% of sand, silt and clay, respectively), the OM content was 2.64% and the field capacity was 21.90% (wet basis). More details about the history of this field can be found elsewhere (Fernández-Bayo et al., 2018; Randall et al., 2020). Dry topsoil was collected from the upper 0–15 cm of the soil in the field site and transferred for further preparations. The soil was sieved (~2 mm) to give unified and homogeneous material. Details about soil amendments characterization and preparation can be found elsewhere (Achmon et al., 2016; Achmon et al., 2017). Briefly, TP was collected from a commercial processing facility during the 2016 harvest season (more details can be found here (Achmon et al., 2018; Achmon et al., 2019)). The TP was sun dried after collection and then stored under ambient conditions. The dried TP was processed in a laboratory blender to reduce the particle size to less than 1 mm to make it unified. Soil was placed inside the mesocosms to achieve 2.5% TP in the soil (on a dry weight basis), a concentration level that was previously shown to be optimal for the CSBS process (Achmon et al., 2018). Non-amended soil was used for SS treatments. Mesocosms containing amended or non-amended soil were transferred to the field site. Mesocosms were constructed by loading soil into 3.8 L plastic soil growth bags (New England Hydroponics, Southampton, MA). The weight of the mesocosms after filling with soil was adjusted to 10 kg (on a dry weight basis) for all systems. All the systems were wetted with distilled water and brought to the field water holding capacity. The mesocosms were laid in pre-dug holes in the field. The field was divided into six plots to give enough material for the greenhouse study and the plots were prepared as previously described (Achmon et al., 2017). Soil treatment was initiated on August 20, 2016 by covering the plot with 0.7 mil, transparent, low density polyethylene film (‘Huskey Film Sheeting’; Poly-America, Inc., Grand Prairie, TX). The experiment lasted 8 days in accordance with previous results (Achmon et al., 2018), after which the plastic film was removed from the field site. The experiment was conducted in proximity (same time and same field site) to other SBS experiments and the data for the treatment temperatures can be found elsewhere (Fernández-Bayo et al., 2018). The mesocosms were left in the field to aerate for additional 15 days to allow for remediation of any residual phytotoxic conditions. After 15 days the mesocosms were transferred to the greenhouse for the growth experiment.

### Greenhouse tomato growth study

Pots (2.37 L) were prepared with the treated soil and were each filled with 6 kg of treated soil for the tomato growth experiment. The pots were assigned randomly to the abiotic stress treatments and were placed on two steel screens metal tables in the greenhouse. The fertigation system was placed prior to transplanting the tomato seedlings the tomato transplants inside the soil. Tomato (cv. SUN6366, Nunhems USA, Inc., Parma, ID) transplants were grown in germination trays in a commercial potting soil mixture (Hastie’s Capitol Sand and Gravel; 25% screened topsoil, 5% lava fines and sand, and 70% mixture in equal parts of forest humus, composted fir, and compost from horse manure and wheat straw). Approximately 2 weeks after germination (BBCH stage 2 digit code 11 or 3 digit code 101), seedlings of approximately the same size were transplanted into pots containing the field soils. The seedlings were transplanted in the soil directly after the field treatment including the aeration stage (at the same day the soil was transported to the greenhouse).

Pots were fertigated twice daily with 300 mL of water containing 143 mg/L N (delivered as 136 mg/L NO_3_ -N and 7 mg/L NH_4_ -N), 63 mg/L P (delivered as H_2_PO_4_), 199 mg/L K^+^, 125 mg/L Ca^2+^, 49 mg/L Mg2+, 65 mg/L S (delivered as SO_4_^−2^), 2 mg/L Fe^3+^, 0.097 mg/L Cu^2+^, 0.633 mg/L Mn^2+^, 0.055 mg/L Mo^6+^, and 0.097 mg/L Zn^2+^. The stress conditions were created as follows: Nitrogen deficiency stress was created by lowering the amount of fertigated nitrogen to 30 mg vs the normal 143 mg (the additional nitrogen coming from the TP was calculated (Achmon et al., 2019) as less than a one dose of 40mg to the samples and hence was considered negligible(. The high salinity stress was created by adding salt as 100 mmol NaCl (A non-lethal concentration stimulates a long term response to salinity and a physiological penalty that was previously used in a relevant study (Zhang and Blumwald, 2001)) to the fertigation regime. Salinity treatments started at 4 weeks after transplanting and continued until harvest. The plants were grown for three and a half months from September 13, 2016 until December 2, 2016 in a semi-controlled greenhouse maintained between 18°C and 28°C and at 50–70% relative humidity with ambient light conditions. The plants were monitored for plant diseases, presence of weeds and fruit ripening.

### Tomato plants analyses

Tomatoes were harvested in a similar way as that of (Achmon et al., 2018) with several modifications. The fruits were picked and counted. The fruits and the plant biomass were weighed (the biomass was dried in oven at 50°C and weighted to determine the dry weight, fruits were weighted on a wet basis). The harvest index was calculated as the proportion of the total fruit weight divided by the sum of the vegetation fresh weight and the total fruit weight. The °Brix was determined using a digital refractometer (PR-100, ATAGO USA, Inc., Bellevue, WA). Measurements of photosynthesis and stomatal conductance were made on fully expanded leaves of plants in proximity to the harvest. Leaves were chosen by leaf number. The 5th leaf (fully expanded) was chosen and a single leaflet per plant was measured. A Li-6400 portable gas-exchange system (LI-COR) was used to measure gas exchange, photosynthesis and stomatal conductance similar to a previous study (Achmon et al., 2018).

### DNA sequencing and tomato metabolomics

The soil microbiome DNA samples were taken from the soil at the harvest from the rhizosphere area of all the treated plants. The DNA was extracted as previously done (Achmon et al., 2020). Briefly, DNA purification was performed using a PowerSoil DNA Isolation Kit (MO BIO Laboratories Inc., Carlsbad, CA). The V4-V5 regions of the 16S rRNAgene were amplified and sequenced using the MiSeqplatform (Illumina Inc., San Diego, CA) in paired-end mode (2 × 300bp read format) with the v3 reagent kit and a qPCR library quantification kit (KAPA Biosystems, Wilmington, MA) was used to determine the concentration of V4 and V5 amplicons capable of being sequenced ahead of loading into the MiSeq system. The complete DNA sequencing procedure was done according to the Joint Genome Institute (Walnut Creek, CA 94598, USA Project ID: 1145678) protocol as described in (Achmon et al., 2020). Metabolomic analysis was done on leaves samples taken from the same leaves that were used for the physiological analysis (described above) All leaflets (including periole/midrib) were sampled and immediately frozen in liquid nitrogen followed by grinding to fine powder. Equal amounts of ground frozen powder were submitted to the West Coast Metabolomics Center (University of California, Davis), extracted, measured, and analyzed by gas chromatography–mass spectrometry (MS) (Gerstel CIS4–with a dual MPS Injector/Agilent 6,890 GC-Pegasus III TOF MS) as described before (Weckwerth et al., 2004)). Processes for the integrated extraction, identification, and quantification of metabolites were performed according to Fiehn et al. (2008). For the extraction, the solvent was prepared by mixing isopropanol/acetonitrile/water at the volume ratio 3:3:2 and degassing the mixture by directing a gentle stream of nitrogen through the solvent for 5 min. The solvent (cooled at −20°C) was added to the ground tissue (1-ml solvent/20-mg tissue), vortexed, and shaken for 5 min for metabolite’s extraction. After centrifugation at 12,800 g for 2 min, the supernatant was concentrated to dryness. The residue was resuspended in 0.5-ml 50% aqueous acetonitrile and centrifuged at 12,800 g for 2 min. The supernatant was then concentrated to dryness in a vacuum concentrator, and the dried extracts were stored at −80°C until use. Untargeted metabolomic analysis was used. The signals were normalized by classic sum normalization i.e. normalization to a sum of intensities in a sample, only that here on the sum was defined as restricted to only identified metabolites to avoid normalizing to peaks that may or may not be possibly related to non-biological compounds (such as phthalates or other laboratory contaminants). This was done with the sum of all peak heights of the annotated detected metabolites as suggested by Fiehn et al. (2008). The equation used in this calculations was (for metabolite i of sample j) metabolite ij, normalized = metabolite ij, raw/mTIC j * mTIC average.

### Data analysis

The microbiome data were initially quantified using QIIME1 (Caporaso et al., 2010). Briefly, the centralized rolling quality control system and the iTagger computational pipeline (Tremblay et al., 2015) for sequence trimming was used. Clustering operational taxonomic units (OTUs) were used based on 97% sequence identity, and taxonomic assignment with SILVA version 119 (Quast et al., 2012). Subsequently, the data were rarefied for diversity analysis (phylum abundance and alpha, beta and gamma diversity) using the ‘phyloseq’ R package. For quantitative analysis, the microbiome count table was normalized using Variance Stabilizing Transformation of the ‘DESeq2’ R package. The physiological and metabolomic parameters were individually compared between solarized and biosolarized treatments using the permuted Brunner Munzel test (*via* the ‘brunnermunzel’ R package). Heatmaps were prepared using the ‘circlize’ and ‘ComplexHeatmap’ R packages, and the boxplots were prepared using the ‘ggpubr’ R package. The raw data of this study can be found in the **Supplementary Materials**.

## Results and discussion

### CSBS impact on tomato plants health under abiotic stress

**Figure 1:**
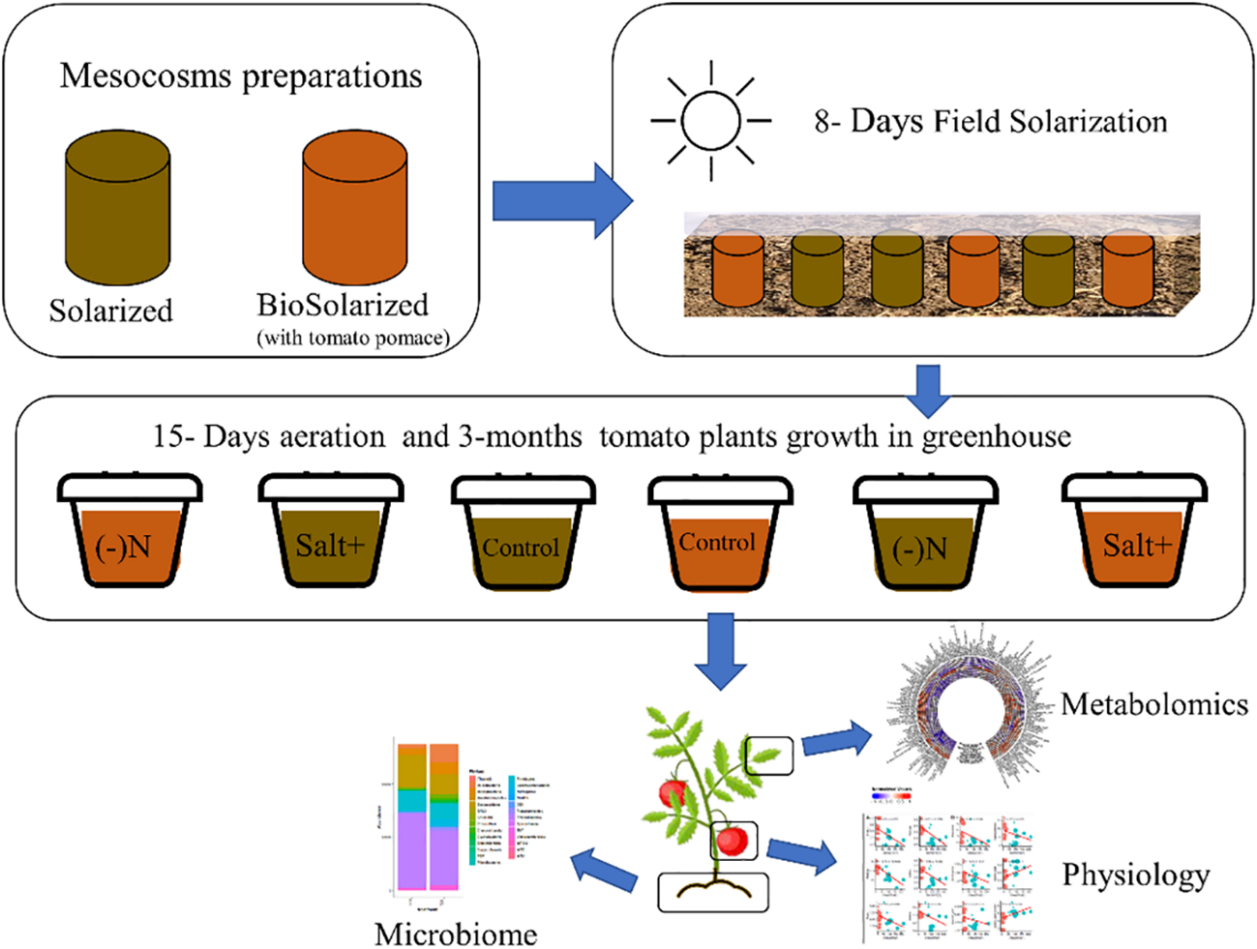
Schematic illustration of the experimental design. Starting by preparing the mesocosms for soil solarization (soil without tomato pomace) and soil biosolarization (with 2.5 w/w tomato pomace). Conducting an 8 days field solarization treatment of the mesocosms. Transferring the treated soil into a controlled greenhouse and transplanting tomato plants under different stress conditions: control – without any stress, salt – adding excess salinity to the soil and Nitrogen deficiency – lower amount of nitrogen in the fertilization regime. After 3-months the plant were harvested and tested for their physiological characteristics (including fruit quality and yield) and leaves’ metabolomics. Additionally the soil was taken for a microbiome analysis.

**Figure 2:**
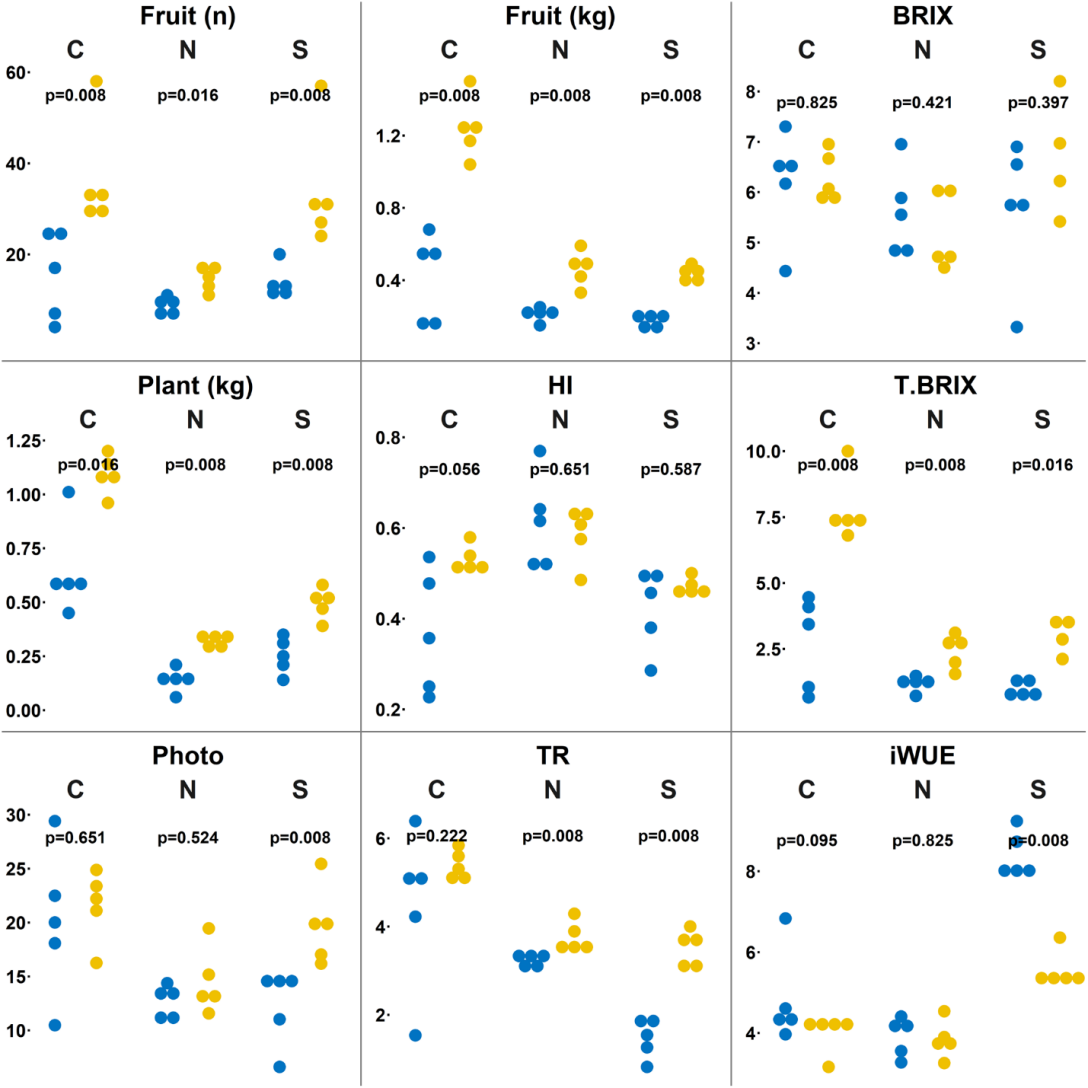
Physiological comparison between solarized and biosolarized pretreated plants. Boxplots of number of fruit (n), plant fresh biomass (kg), fruit weight (kg), harvest index in proportion of fruit weight out of total weight (HI), sugar content (BRIX), Total sugar content as the product of fruit weight and sugar content (T.BRIX), Leaf gas exchange analysis as carbon dioxide assimilated (Photo), transpiration as water transpired over time and space (Tr), instantaneous water use efficiency (iWUE) comparing solarized (SOL) and biosolarized (BIO) pretreated plants under well-watered (C), nitrogen-deprived (N) and salinity-treated (S) (n=4-5) plants. Significance was tested using the permuted Brunner Munzel test, presenting the p-values.

This study was designed (as shown in **Figure 1**) to explore the effects of a short time CSBS with TP residues on the health of tomato plants (including a deep look into the metabolome of the plants’ leaves) and of the soil microbiome under abiotic stress of nitrogen deficiency and high salinity. The study was designed to compare the CSBS with traditional SS. Previous studies (Achmon et al., 2017; Achmon et al., 2018) showed that CSBS and SBS can be effective in terms of inactivation of weeds and pests even after a short treatment of less than two weeks (whereas SS usually lasts at least one month or more (Achmon et al., 2017)). Yet, the impact of SBS and CSBS under abiotic causes of stress such as soil salinity, drought and lack of sufficient soil nitrogen has not been previously explored. In this study the treatment of 8 days solarization was done in field conditions prior to the use of the soil as potting soil in the greenhouse study. This was done to mimic the solarization treatment in an optimal way while at the same time enabling a specific look per plant per treatment in the greenhouse. In a previous study it was found that a 12 days remediation of the soil can be too short and can cause some lingering residual phytotoxicity in the soil (Achmon et al., 2018). To avoid phytotoxicity all samples were given a 15 days remediation period. After the remediation period the tomato plants were grown in different conditions of salinity and nitrogen deficiency (as described above in the methods section) for a period of three months until the harvest. The results indicate that CSBS was superior (P<0.05) to SBS in this study in terms of total yield, fruit numbers, plant weight, and plant health (carbon dioxide assimilation, water transpiration) under both abiotic stresses and the control (no stress) treatments (**Figure 2**). Interestingly, the total BRIX per plant (representing the total potentially sugar content a plant can produce, an important parameter for processing tomatoes; (Gur et al., 2011) was higher in all CSBS treatments as compared to SS. These findings support several studies that showed that SBS can have an advantage in plant health and crop yield in addition to its effect against weeds and pathogens (Domínguez et al., 2014; Chamorro et al., 2015; García-Raya et al., 2019). However, this is the first study that showed a beneficial effect of CSBS in conditions of abiotic stresses. It is noteworthy to note that the effect under salinity conditions was such that CSBS did as well as, and even better than, the control SS treatment (**Figure 2**). To better understand the alleviation of CSBS effects in abiotic stresses an examination of the plant leaves metabolome and soil microbiome was done.

### CSBS impact on tomato plants metabolome under abiotic stress

**Figure 3:**
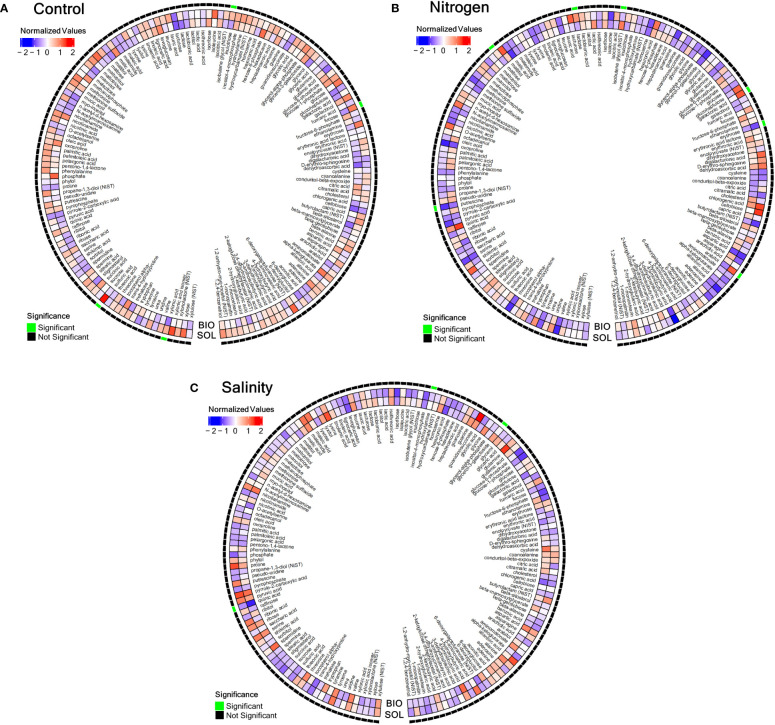
Metabolomic comparison between solarized and biosolarized pre-treated **(A)** control-treated plants, **(B)** nitrogen-deprived plants, **(C)** salinity-treated plants. A heatmap comparing the standardized averages of metabolite counts in salinity-treated solarized (SOL) and biosolarized (BIO) plants (n=5). Each metabolite value was standardized as the standard deviation from the average metabolite value of all plants (n=30), and the values were averaged for solarized and biosolarized treatments and were shown in the red-white-blue scale (blue denotes low values and red denotes high values), while significance (p<=0.05) was tested between the two groups (n=5) using the permuted Brunner Munzel test (significance was denoted by an outer green box, while insignificance was denoted by an outer black box). values), while significance (p<=0.05) was tested between the two groups (n=5) using the T-Test (significance was denoted by an outer green box, while insignificance was denoted by an outer black box).

The analysis of the metabolites of the leaves of the tomato plants was done in close proximity to the harvest to reflect the plant health at the same conditions as those of the tomato plants’ fruits and the soil microbiome. The metabolomic analysis showed significant differences in the control treatment between the CSBS and the SS in terms of inulotriose, xylonic acid, sulfuric acid and fucose (**Figure 3A**). These sugars and acids have roles in important cellular processes such as the biosynthesis of starch and other energy storing molecules (Preiser et al., 2020), and in stress tolerance (Carrasco-Gil et al., 2021) and they might be connected to defending mechanisms against fungal attacks (Bashir et al., 2016). In samples of nitrogen deficiency the differences in metabolites were also mainly in sugars. The significant differences (P<0.05) were found in lactose, putrescine, arabitol, maltose, isobutene glycol, arabitol, galactonic acid and erythrose (**Figure 3B**). Putrescine was previously recognized as a metabolite with a role in stress responses in different types of abiotic stresses in tomato such as chilling tolerance (Song et al., 2014), drought (Farzane et al., 2020), and also nitrogen deficiency (González-Hernández et al., 2022). Some of the sugars identified as present in cases of significant difference in nitrogen deficiency conditions between CSBS and SS are associated with ripening processes in tomato fruits (Badejo et al., 2012). In the salinity stress test the two metabolites that showed significant differences between the three treatments were quinic acid, glycerol-3-galactoside and hydroxylamine (**Figure 3C**). Quinic acid was previously associated with salinity stress in tomato plants (Kwon et al., 2019; Moles et al., 2019) and other plants (LI et al., 2021), but the exact mechanism of its association with the stress is unclear. Glycerol-3-galactoside and hydroxylamine were not been known previously to be related to salinity stress in tomato plants, but hydroxylamine as it is an important metabolite in nitrogen cycles in the plant (Yao et al., 2022) it can be hypothesized that its role in stress can be connected with protein synthesis in the plants. The metabolomic results in this study indicate that CSBS can have a specific impact on the plants metabolism. It is also important to note that in both the control group and the nitrogen deficiency group the main differences were mostly noted on sugars. This result should be further explored in future studies. Additionally, it is important to mention that some metabolites such as volatile organic compounds were not within the focus of this study. These metabolites can also have a significant contribution to the observed results and should be investigated in a future study as well. In this study the assumption was that the impact on the plants metabolism will be associated with changes of the microbial population (e.g. microbiome) in the soil rhizosphere. To elucidate these changes the soil rhizosphere at the time of harvest was taken for DNA sequencing.

### CSBS impact on the soil microbiome under abiotic stress

**Figure 4:**
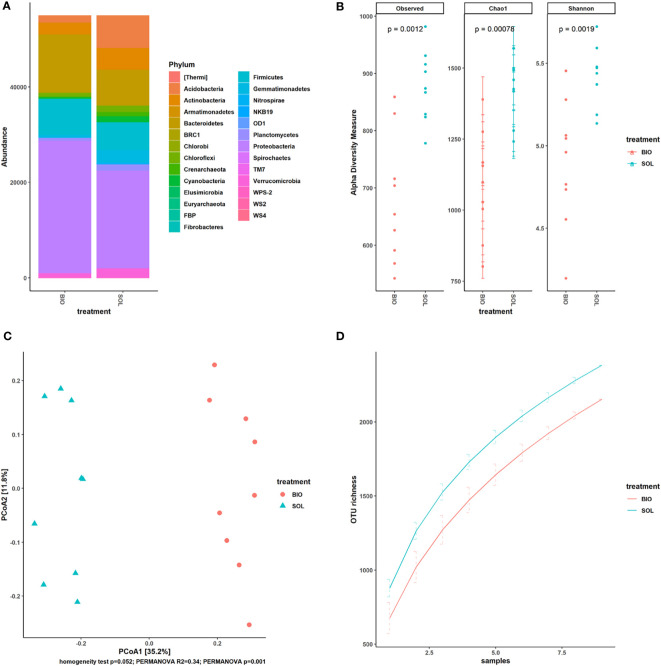
Microbiome diversity comparison between solarized and biosolarized pretreated plants. OTU counts of solarized (SOL) and biosolarized (BIO) plants (n=18) were rarefied. **(A)** comparison of the abundance of taxa by phylum (n=9). **(B)** Alpha diversity analysis using observed species, Chao1 and Shannon indices together with p-values (significance was tested using an unequal-variance Mann-Whitney U test). **(C)** Beta diversity analysis with Principal Coordinates Analysis (PCoA) using Bray-Curtis dissimilarity. Significance was tested *via* PERMANOVA. **(D)** Gamma diversity analysis using geocluster accumulation curves for mean OTU richness from a random sampling of geoclusters ± SD.

Several recent studies of SBS showed the impact this agricultural technique has on the soil microbiome (Achmon et al., 2017; Achmon et al., 2020; Fernandez-Bayo et al., 2020; Randall et al., 2020). Across all these studies, SBS was able to alter the soil microbiome significantly. Our results showed, as the previously mentioned studies suggested, that CSBS had a different impact on the soil microbiome than the SS treatment (**Figure 4**). CSBS elevated the abundance of Firmicutes, similar to the trend that was shown in other recent studies (Achmon et al., 2020; Shea et al., 2022) (**Figure 4A**). This might suggest that the soil conditions developing in SBS are more anaerobic than those in SS treatment, a finding that was also previously reported (Achmon et al., 2018). Changes in the abundance of Proteobacteria and Bacteroidetes were also noticeable and in a same trend in a similar CSBS (Achmon et al., 2020). Interestingly, the diversity was significantly lower in the CSBS than in the SS (**Figure 4B**) and also the richness (**Figure 4D**), which can be expected as the CSBS treatment applies additional natural selection forces because of the high volatile organic compounds produced which are mainly volatile fatty acids (Hestmark et al., 2019; Fernandez-Bayo et al., 2020; Liang et al., 2022).

### Relationships between microbiome metabolome and plant physiology in CSBS done under abiotic stress

**Figure 5:**
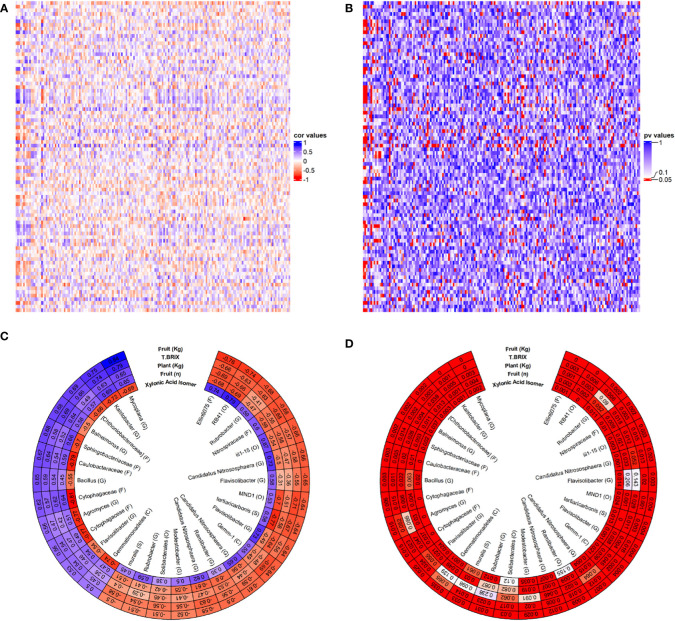
Correlations between the microbiome and between physiological and metabolomic measurements. OTU count data were normalized using Variance Stabilizing Transformation and OTUs with missing data (NAs) were filtered out. **(A, B)** Each OTU was correlated (Spearman) with each physiological measurement and metabolite and correlation values **(A)** and p-values **(B)** are presented. **(C, D)** The most highly correlated OTUs and metabolomic and physiological measurements were manually sifted and the correlation values **(C)** and p-values **(D)** are presented.

This study is the first to date to correlate between the microbiome of CSBS/SS (or SBS) and tomato plants metabolome and physiology. The leaves were chosen as the metabolomic focus of this study in order to try to examine the correlations between the soil microbiome below the ground, that is directly impacted by the soil treatments and the abiotic stresses, and the above ground plant growth which is indirectly impacted by the soil treatments and the abiotic stresses. The result of this correlation is shown in **Figure 5**. The approach in this study was to take the array of data (e.g. metabolites+OTUs+physiology) of all the tests together and to try to sieve out the most significant one. This approach was taken since no specific prior knowledge about this system is available and to avoid imposing biased knowledge on the data. **Figures 5A, B** show the results of the total correlations between the leaf’s metabolites and physiological characters of the tomato plants under the different conditions and the different OTUs of the soil microbiome. The results showed many positive and negative correlations (**Figure 5A**) most of which were significant (5B). To get more insight from the data, an additional filtration process was imposed to get the most significant OTUs and plant characteristics. The filtration process that was chosen was characteristics and OTUs combination which has an above 80% significant P values combined with R>0.5 or R<-0.5. **Figures 5C, D** present the results of the filtration process for the 30 OTUs and plant characteristics that were filtered from the cut-off. Interestingly, only one metabolite and four tomato plants physiological characteristics were filtered in. The metabolite that showed a significant correlation with these OTUs was xylonic acid isomer. The xylonic acid isomer was positively correlated with R>0.7 with the OTUs of: Ellin6075, RB41, *Candidatus Nitrososphaera* and *Flavisolibacter*. The OTUs with a negative correlation of R<-0.7 to the Xylonic Acid isomer were: *Kaistobacter*, Sphingobacteriaceae, Caulobacteraceae, Cytophagaceae, *Agromyces*, Cytophagaceae and Gemmatimonadetes. Although xylonic Acid is not known to be directly connected with SBS or with abiotic stress it is a degradation product that is derived from xylem and is produced by microbial metabolism (Zhang et al., 2017; Trichez et al., 2022). The OTUs in this study are not known to be associated with xylonic acid metabolisms and additional studying is needed to try to understand the rule xylonic acid may play in SBS systems. It is worth noting that the Acidobacteria RB41 which had the highest positive correlation with the xylonic acid isomer, was found to have a key role in control over soil carbon cycle (Stone et al., 2021). The main interesting finding about the xylonic acid isomer is the fact that it is the exact mirror image of the wanted physiological characteristics in the tomato plants. This might suggest that the xylonic acid isomer can be used as a negative indicator for the success of SBS. Unlike the leaf’s metabolites, four physiological characteristics (fruit weight, total Brix, plant weight and fruit number) were all filtered in by the cut off, with highly significant correlations with 30 relevant OTUs (**Figures 5C, D**). It is important to note that all these characteristics showed significant differences between SS and SBS treatments (**Figure 2**) and they were shown here again by using a non-supervised statistical tool. The fruit weight was highly correlated positively (R>0.7) with *Mycoplana* and *Kaistobacter* and was negatively correlated with Ellin6075 and RB41 (R<-0.7). *Mycoplana* was reported to have some role in bioremediation of soils (Cao et al., 2018; Wolf et al., 2019) and is showing an elevated abundance in these studies, which may indicate its potential in bio degradation of organic material such done in the case of SBS. More importantly, some of the *Mycoplana* species were found to be growth promoting bacteria found in wheat in nutrient-poor Calcisol and have some antifungal activity in plant diseases (Legrand et al., 2019). These attributes might explain the positive correlation *Mycoplana* has between the fruit weights and number, total brix and plant weight (**Figure 5C**) in this study. Additionally, it was found *Mycoplana* has a role in salt stress plant growth (Egamberdieva et al., 2018), suggesting that salt stress in this study can also cause the elevated abundance of *Mycoplana*. Similar trend as that found in *Mycoplana* was also found in *Kaistobacter* which was also found to be a potential disease suppressing bacteria in plants (Liu et al., 2016). *Kaistobacter* species are also involved in the soil bioremediation process (Gao et al., 2021; Lin et al., 2021). This might suggest that bacteria species that are adapting fast to harsh contaminated environments might also adapt fast to “organic” contaminations such as the one in SBS. These findings is suggesting that SBS might has also the potential to be effective in solving cases of other soil stressors such as heavy metals’ contamination, but this was not within the focus of this research and should be tested in a future study. While *Mycoplana* and *Kaistobacter* can have the potential to serve as positive indicators for the SBS process success on tomato plants consecutive growth. Ellin6075 and RB41might serve as negative indicators for SBS as they were negatively correlated with all the plant’s four physiological characteristics described above. The Ellin6075 bacterial family was previously found to be correlated with the soil’s location and with using a no-till practice (Yin et al., 2017), but we have found no indication in the current literature for any role in soil stresses. As Ellin6075 bacterial family has some role in organic matter decomposing (Ye et al., 2016) and it is might be worth to focus on this family in future SBS microbiome studies. The RB41 bacterial order has previously shown an elevated abundance in soil under stress, including a salinity stress (Wang et al., 2019). It might be the case that the RB41 bacterial order changes of abundance is more related to the stress conditions than to the SBS treatment, but this should be further explored in the future. Except of Ellin6075 and RB41OTUs the OTUs of MND1 and *tertiaricarbonis* also showed highly negative correlation with the plants’ four physiological characteristics. The MND1 bacterial order was also found in several studies that were looking at contaminated soils (Shen et al., 2018; Wang et al., 2020) suggesting that this order can be fast adapted to harsh conditions in the soil and is maybe an indication for the existence of abiotic stress. *Tertiaricarbonis* species negative correlation in this study with fruit weights and number, total brix and plant weight is interesting as it is not known to be related to soil stress or as a negative indication for soil conditions or for plant growth. *Tertiaricarbonis* is usually coming from aquatic systems (Chen et al., 2018; Brand et al., 2019) and is worth an additional study to see if it has a role in SBS systems. The result of the correlation between the microbiome and the plant characteristics in this study suggest that the soil microbiome has a significant impact on the plant growth performance in tomato CSBS treatments.

## Conclusions

This is the first study that looked at the impact SBS has on consecutive plant crops under abiotic stress conditions. The results showed that CSBS with TP can alleviate the damaging effect of abiotic stresses in cases of high salinity and nitrogen deficiency compared to the traditional SS technique. The impact on soil microbiome and plant metabolome is also significant in CSBS and can partially explain the advantage that CSBS has compared to SS. The results suggest that several OTUs have important role in CSBS performance in tomato growth. *Mycoplana* and *Kaistobacter* genera showed potential to serve as positive indicators for successful CSBS and on the contrary Ellin6075, RB41, MND1 and *tertiaricarbonis* might serve as negative indictors. Additionally, xylonic acid isomer metabolite in the plant leaf was highly correlated with poor plant agriculture performance in this study and should gain more attention in future SBS research. This study is an important step towards the implementation of SBS techniques as a broad group of soil treatments that can be used to mitigate a wide variety of agricultural problems and doing so by creating additional solutions of getting rid of organic waste residues. As this is the first time SBS was tested as a solution for growing plants under abiotic stress, further studies should be done with different stressors and different organic amendments as well as large scale field experiments to explore the full potential this of treatment and the microbiome landscape involved in this response.

## Data availability statement

The data presented in the study are deposited in the DOE Joint Genome Institute repository, accession number 1145680. https://genome.jgi.doe.gov/portal/Soiandcoitagspl5_FD/Soiandcoitagspl5_FD.info.html.

## Author contributions

YA and NS conceived of the presented idea. ZH, DT performed the computational analysis on microbiome and metabolomics, MMRW and NS conducted the greenhouse experiments, JF-B, DH and YA conducted the field experiments, ZH, JJS, JV, EB, CS, NS and YA wrote the manuscript. All authors contributed to the article and approved the submitted version.

## Funding

This study was partially supported by a grant from the National Institute of Occupational Safety and Health (grant agreement number U54 OH007550) and California Department of Pesticide Regulation (grant agreement number 14-PML-R004). YA is partially support by the Guangdong Provincial Key Laboratory of Materials and Technologies for Energy Conversion, by the 2021 Guangdong Special Fund for Science and Technology, Multi-effect valorization of tea waste by soil biosolarization and restoration of farmland soil ecosystem (#STKJ2021128) and Special funds for higher education development of Guangdong Province. ZH was partially supported by a scholarship from The ADAMA Center for Novel Delivery Systems in Crop Protection, Tel Aviv University.

## Conflict of interest

The authors declare that the research was conducted in the absence of any commercial or financial relationships that could be construed as a potential conflict of interest.

## Publisher’s note

All claims expressed in this article are solely those of the authors and do not necessarily represent those of their affiliated organizations, or those of the publisher, the editors and the reviewers. Any product that may be evaluated in this article, or claim that may be made by its manufacturer, is not guaranteed or endorsed by the publisher.
