# Supplementary figures and images for: The effect of circular soil biosolarization treatment on the physiology, metabolomics, and microbiome of tomato plants under certain abiotic stresses
Source: Front Plant Sci. 2022 Nov 8;13:1009956. doi: 10.3389/fpls.2022.1009956 (PMC9679285; doi:10.3389/fpls.2022.1009956)

## Slide 1
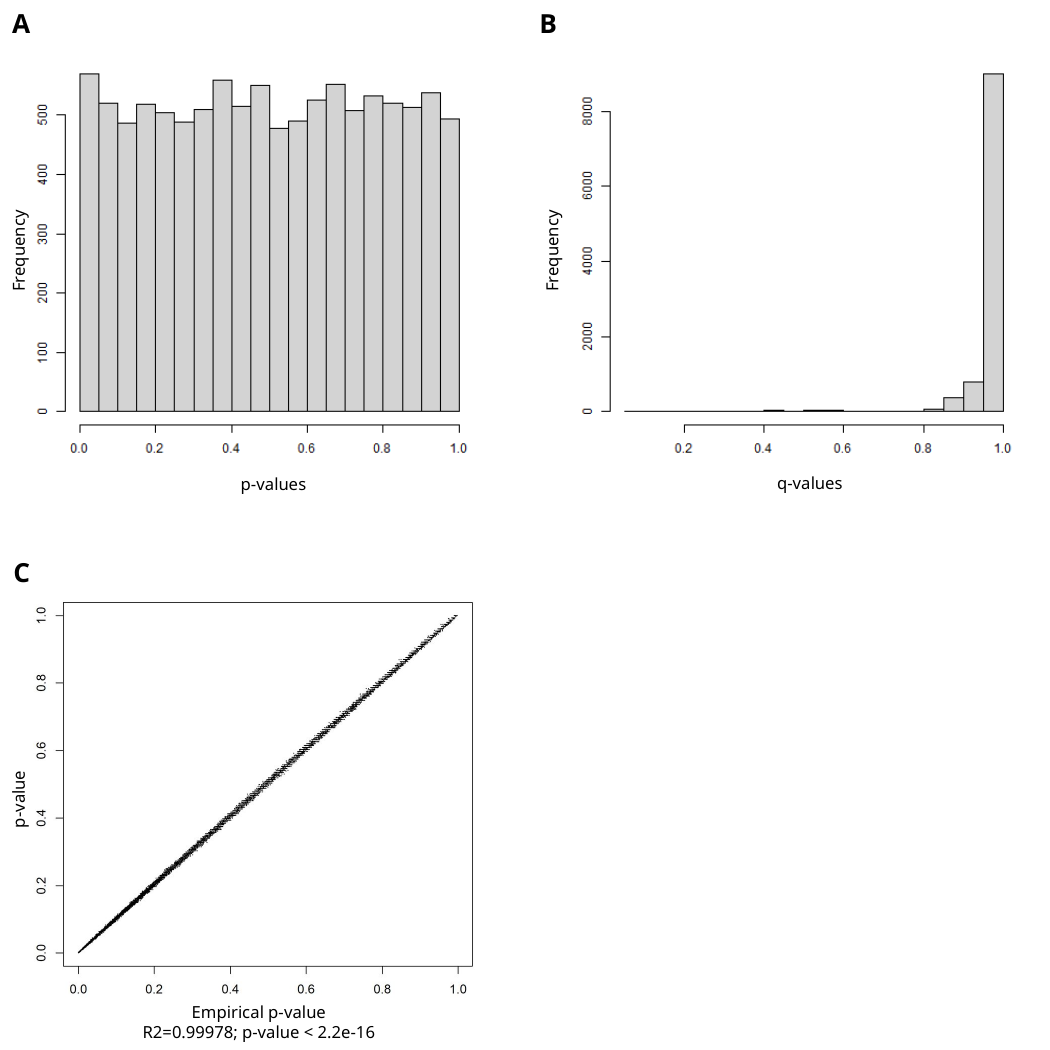

A
Frequency
p-values
B
Frequency
q-values
C
p-value
Empirical p-value
R2=0.99978; p-value < 2.2e-16

Supplement: Supplementary file 1 [file DataSheet_1.zip › supp. tables&figures fpls/Supp. Fig. 1.pptx]

## Slide 1
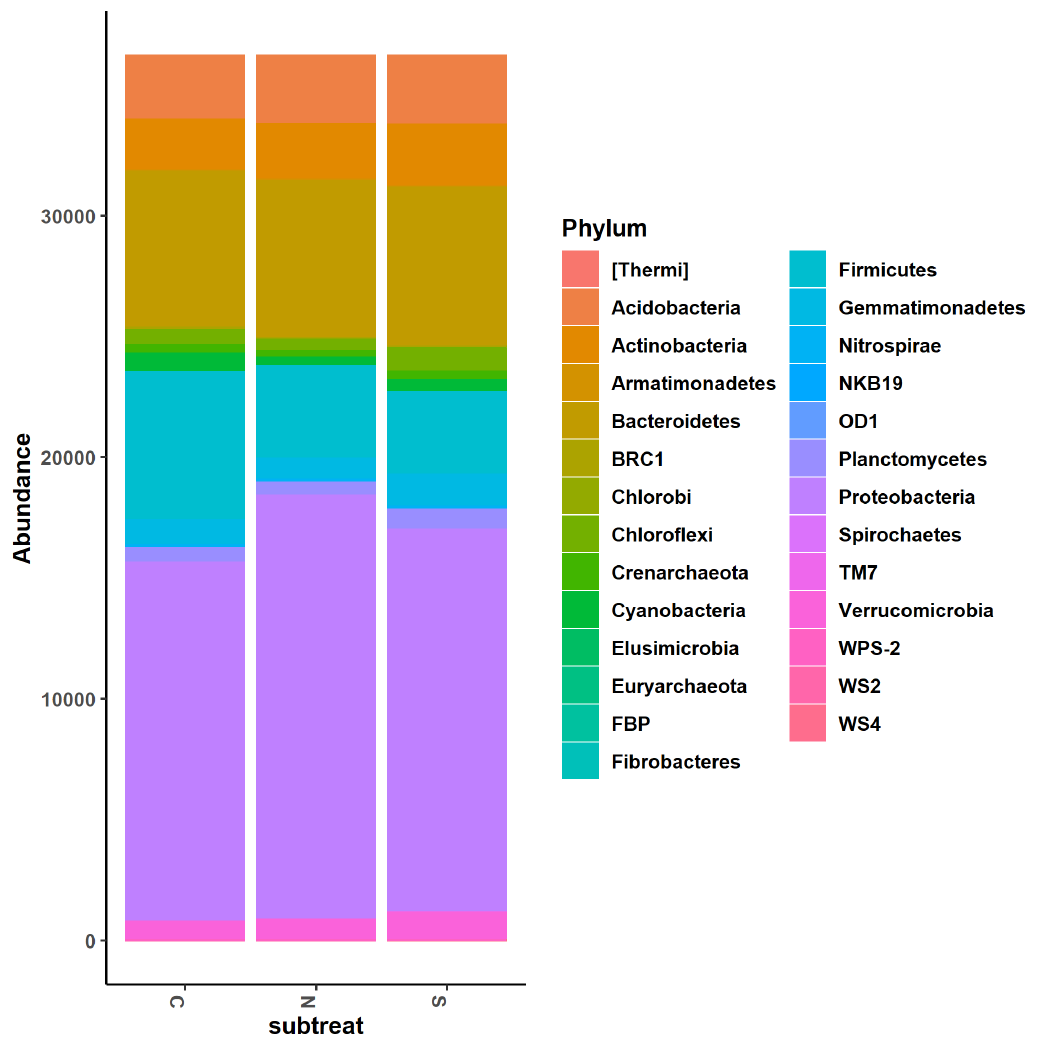

Supplement: Supplementary file 1 [file DataSheet_1.zip › supp. tables&figures fpls/Supp. Fig. 2.pptx]
